# Outcomes in Cirrhosis-Related Refractory Ascites with Emphasis on Palliative Care: Single-Centre Experience and Literature Review

**DOI:** 10.1007/s11901-024-00669-0

**Published:** 2024-04-02

**Authors:** Marcus Rex English, Jordache Ellis, Sumita Verma, Yazan Haddadin

**Affiliations:** 1 Department of Gastroenterology and Hepatology, University Hospitals Sussex NHS Foundation Trust, Brighton, UK; 2 Department of Clinical and Experimental Medicine, Brighton and Sussex Medical School, North South Road, Falmer, Brighton BN1 9PK, UK

**Keywords:** Recurrent ascites, Advanced chronic liver disease, Long-term abdominal drains, Quality of life, Liver transplantation, Large-volume paracentesis

## Abstract

**Purpose of Review:**

Despite refractory ascites (RA) due to cirrhosis having a median transplant-free survival of 6–12 months, palliative care (PC) input remains uncertain. We aimed to review the existing literature on clinical outcomes in cirrhosis-related RA and report the findings of a single-centre retrospective cohort study with a special focus on linkage to PC in this cohort of patients.

**Recent Findings:**

Our study and subsequent literature review confirm the high mortality associated with cirrhosis-related RA (19–55% 1-year mortality) with only a minority of patients receiving curative options (3–23%). Despite this, in our study only a minority of patients (33%) were referred to PC. None of the studies identified in the scoping review makes any references to palliative care use.

**Summary:**

Our own data and a literature review confirm that, despite high mortality, only a minority with RA due to cirrhosis are referred for specialist PC input and often too late in their disease trajectory. Future research should focus on patient-centred outcomes in this cohort of patients where optimising quality-of-life and facilitating advanced care planning should be a priority.

## Introduction

Liver-related deaths in England have increased by more than 250% since 1971, and now constitute the fourth commonest cause of premature death [1]. Ascites is the most common complication of cirrhosis (with approximately 40% of patients developing it within 5 years) and the most frequent cause for hospitalisation [2, 3]. Refractory ascites (RA), defined by intolerance or unresponsiveness to diuretics [4, 5], is a useful prognostic indicator as median transplant-free survival is about 6 months [6–9]. Although liver transplant can be curative, demand for organs continues to outstrip supply [10, 11]. The transplant process involves major surgery and life-long immunosuppression, meaning many patients with significant comorbidities or frailty are not potential candidates [12]. Similarly transjugular intra-hepatic portosystemic shunts (TIPS) can be associated with improved survival but carries procedural risks and possible complications including worsening hepatic encephalopathy [13, 14•]. As a result, a significant proportion of patients with RA are reliant on symptomatic relief through repeated hospitalisation for large-volume paracentesis (LVP) [14•, 15]. Alternative management options include automated low-flow ascites pump (alfa pump) and palliative long-term abdominal drains (LTADs), with potential benefits to patient care [16, 17•].

Ascites (including RA) is associated with a high symptom burden and poor health-related quality of life (HRQoL) [18–21]. Although RA secondary to cirrhosis is being recognised as a potential trigger for palliative care (PC) involvement, there remains paucity of published data with researchers focused primarily on clinical outcomes and the impact of therapeutic interventions on survival [22]. For example, a recent systematic review included 77 studies assessing TIPS in patients with RA even though the majority of these patients will not receive one [14•]. There is a need to shift the focus of research in this group of patients living with this serious life-limiting illness. The aim of this manuscript therefore was to conduct a scoping review assessing outcomes in RA with emphasis on linkage to PC and provision of advanced care planning (ACP). We then conducted a retrospective cohort study in our own centre with a specific focus on PC provision.

## Scoping Review Methodology

Eligible studies were defined as any non-interventional, cohort study or case series that reported on clinical outcomes of patients with RA secondary to cirrhosis. Studies had to have a clear definition of RA and report the outcomes of this cohort of patients separately. Authors of studies that included patients with RA but did not report on their outcomes were contacted for the raw data. Studies were identified by conducting a PubMed literature search using the following terms: (refractory ascit*) AND (cirrho*) AND (outcomes OR mortality OR survival).

## Scoping Review Results

Six hundred seventy-four titles were reviewed of which 12 studies met our inclusion criteria. Of these 12 studies, one was excluded as the manuscript was in French and four as the authors did not provide the raw data for the cohort with RA. Seven studies were therefore included in the final analysis and data extracted are summarised in Table 1.

Only four studies were published in the last 5 years. Six studies used the International Ascites Club (IAC) definition for RA in their inclusion criteria, but the methodology in establishing true resistance or intolerance was variable (as expected with the retrospective nature of the studies). One was published before the IAC definitions were established in 1993. The number of patients with RA varied between 24 and 241. One-year mortality was reported in six studies and ranged between 19.2 and 55%. Rates of transplant (1.7–23.2%) were reported in six studies and TIPS (1.0–4.3%) in two.

None of the studies looking at patients with RA mentioned or acknowledged the importance of PC.

## Retrospective Cohort Study

### Patients and Methods

Our retrospective cohort study was conducted in a secondary care 1100-bedded hospital in southeast of England. All patients coded as having a diagnosis of ascites from 1st of June 2012 to 30th June 2019 were identified by the clinical coding department and electronic hospital records. Initial records (discharge and clinic letters) were screened by MRE and in case of any uncertainty advice was sought from SV.

### Inclusion Criteria

RA as defined by IAC criteria [5]. Patients had to have a documented diagnosis of RA made by a gastroenterologist, or three or more LVPs during the study period as a proxy for diuretic intractable ascites.

### Exclusion Criteria

Non-cirrhosis cause of ascites and/or if aetiology of cirrhosis uncertain

Incomplete medical records

Ascites occurring after liver transplantation

Baseline clinical and demographic data were extracted from electronic discharge summaries and clinic letters at the time of RA onset and entered onto an anonymised database. These included the aetiology of liver disease, age, sex, use of diuretics, biochemical data, liver prognostic scores, ascitic fluid protein, past medical history including spontaneous bacterial peritonitis (SBP) and antibiotic prophylaxis. Comorbidities were categorised as follows: gastrointestinal disease (e.g. inflammatory bowel disease, pancreatitis), cardiovascular (e.g. hypertension, cerebral vascular accidents and heart failure), respiratory (e.g. chronic obstructive pulmonary disease, asthma or obstructive sleep apnoea), psychiatric (depression, anxiety, PTSD, psychosis), renal, neurological, non-HCC malignancy, diabetes, non-diabetic endocrinology (e.g. hypothyroidism, osteoporosis), infectious diseases and a category of ‘other’.

Study outcomes included referrals to PC and presence of documented end-of-life discussions. Clinical outcomes included number being referred to and receiving TIPS/transplant, number of LVPs and predictors of mortality. Time between RA diagnosis and referral to PC and the time between PC referral and death were also looked at.

Follow-up data was collected until 31st of March 2020 after which patients were censored. This date was chosen to avoid any potential impact that COVID-19 pandemic might have had on clinical outcomes.

### Statistical Analysis

Data are summarised using counts, means ± standard deviations, medians (interquartile ranges [IQR]) or percentages. Potential predictors of mortality were defined a priori. These included age, gender, CPS, MELD-Na and UKELD scores; diuretic use, SBP incidence, comorbidity, TIPS/transplant or LTAD insertion. Variables associated with mortality on univariate analysis were included in a multivariable logistic regression analysis. Statistical analysis was performed using Stata 17.

### Ethical Approval

As this study was an anonymised retrospective audit, formal ethical approval/informed consent was not deemed necessary by our Research and Development Department.

## Results

Of the 1536 patients initially identified as having ascites, 869 were excluded due having a non-cirrhotic cause or incomplete medical records. Of those with ascites due to cirrhosis (*n* = 667, 43%), *n* = 88 (13%) meet our criteria for RA and were included in the final analysis. Baseline demographic data of the cohort is summarised in Table 2.

This was a relatively young and predominantly male cohort with over two thirds being on diuretics and just under 40% having a prior history of SBP. The predominant aetiology of cirrhosis was alcohol-related liver disease (ALD) in just over 80%. The three most common comorbidities were cardiovascular, diabetes mellitus and psychiatric illnesses (Table 2).

At the time of RA diagnosis, median CPS was 9, while mean MELD-Na and UKELD scores were 17 ± 7 and 55.7 ± 5.2, respectively. About a third had hyponatraemia and/or renal impairment.

The median number of LVPs was 5 (IQR 3–8), suggesting high attendance rates to ambulatory care services. Six patients had over 20 LVPs each. Palliative LTADs were inserted in 11 (12.5%) patients.

### Referral for Liver Transplant and TIPS

Overall, 31 patients (35%) were referred for liver transplant and six (7%) for TIPS. Eventually only 11 (13%) patients received a transplant, and one patient received a TIPS (1%). Outcomes are summarised in Table 3.

### Palliative Care Input

Although 56% (*n* = 49) of patients had documented end of life care discussions, only 33% (*n* = 29) were referred to palliative care. The median time from RA diagnosis to PC referral was 15 weeks (IQR 4–32), time from PC referral to death being 9 weeks (IQR 4–16) (Table 4). Independent predictors of referral to PC were LTAD insertion (OR 11.0, CI 2.11–57.0, *p* < 0.01) and age (OR 1.57/10 years, CI 1.04–2.38, *p* = 0.03). There was a trend for PC referral to be a predictor of death outside hospital, though this failed to reach statistical significance (OR 2.83, CI 0.89–9.04–0.89, *p* = 0.078).

### Mortality

**Fig. 1:**
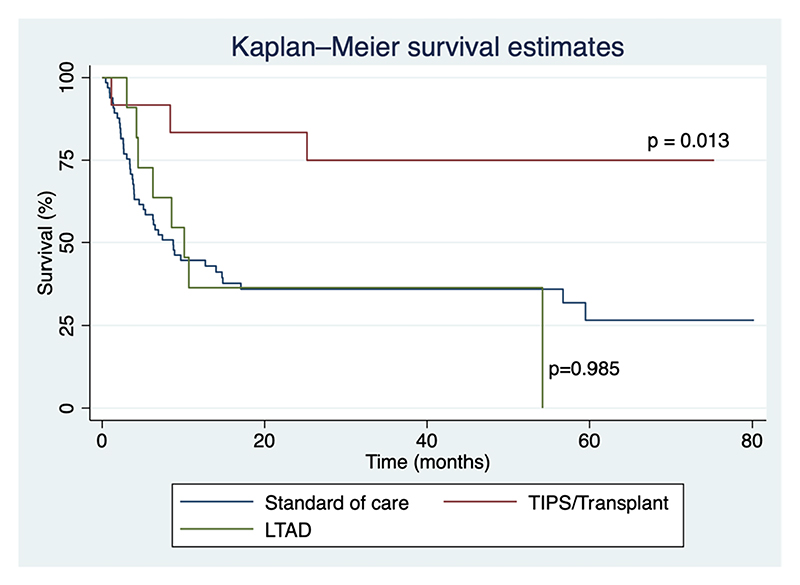
Kaplan–Meier survival in patients with refractory ascites who underwent liver transplant/TIPS, long-term abdominal drains or standard of care (large-volume paracentesis)

Over a median follow-up of 298 days (IQR 115–125), the overall mortality was 61%, 1-year mortality being 51%. Compared to those receiving standard of care (LVP), receiving transplant/TIPS was associated with a significantly higher 1-year survival (43% vs. 83%, *p* = 0.013). Compared to those receiving standard of care (LVP), receiving LTAD was not associated with a 1-year survival benefit (43% vs. 36%, *p* = 0.985). Kaplan–Meier survival curves for each group are shown in Fig. 1.

Univariate and multivariate analysis was performed to assess predictors of mortality (Supplementary—Table 4) Independent predictors of mortality were protective effects of undergoing transplant/TIPS (OR 0.15, CI 0.04–0.64, *p* = 0.01) and having a psychiatric comorbidity (OR 0.32, CI 0.11–0.97, *p* = 0.044).

## Discussion

Our retrospective cohort study and scoping review confirm the poor prognosis associated with refractory ascites secondary to cirrhosis. Our 1-year transplant free survival was 43%, with only a minority receiving curative options. Our mortality rates were higher than some of the studies identified in our scoping review [23, 24] but consistent with those that used more stringent criteria to diagnose RA (IAC) [5, 8, 25, 26].

Despite the high mortality associated with RA, none of the studies identified in the scoping review assessed PC input in this cohort. It is disappointing to note that even in our retrospective study, only a third were referred to PC services, most dying within 2 months of the referral. Our data is consistent with earlier studies that PC delivery in advanced cirrhosis is often too little, too late [27–29].

PC is specialised medical care for people living with a serious illness. It focuses on relieving symptoms and stress of the illness, thus improving HRQoL for patients and their families [30]. PC can mistakenly be considered synonymous with end-of life-care. Additional misconception amongst clinicians is that PC necessitates the end of active treatment. However, the modern PC model involves holistic care delivered to patients with a serious illness at any point in their clinical trajectory, including alongside curative treatments [31]. While the precise optimal timing of PC referrals remains controversial, early PC in advanced cancer does appear to improve quality of life and symptom burden [32, 33] and enables acceptable goals and advance care planning to be established. This might be beneficial in patients with RA who have a significantly reduced life expectancy [34].

Approaching challenging end-of-life care discussions in the context of a fluctuating disease trajectory in patients with ACLD can reduce physicians’ confidence in referring to PC [35, 36]. There may also be a perception that PC equates with excluding patients from receiving a liver transplant [37]. Additionally, physicians may have limited awareness of the poor prognosis of RA [29]. Indeed, as our retrospective study has demonstrated, traditional prognostic scores such as CPS, MELD-Na and UKELD were not predictors of mortality in patients with RA. Finally, potential barrier to PC provision in RA is the paucity of evidence-based palliative interventions in advanced chronic liver disease (ACLD).

Encouragingly, there is now increasing recognition of the need for evidence-based palliative interventions in ACLD, supported by national and international guidance [38, 39•]. This has led to randomised controlled trials (RCT) such as the PAL-LIVER study [40] and the advance care planning video study [41]. An earlier feasibility RCT showed preliminary evidence of safety and efficacy of palliative LTADs in RA due to cirrhosis [17•], leading to an ongoing national multicentre RCT (REDUCe 2 Study) assessing this intervention in RA [42]. Additionally, ‘gatekeeping’ needs to be addressed [43] as well as improving communication skills. There also needs to be increased emphasis on PC both at undergraduate and post graduate level. Finally, bringing together multidisciplinary colleagues (British Association for Study of Liver Disease End of Life Specialist Interest Group) [44] can only be a step in the right direction.

Our study does have limitations. Given its retrospective nature, no patient-reported outcomes are available to assess the impact of the PC intervention. There would have also been potential under reporting of both RA development as well as the PC intervention itself. Additionally, some of the results might not reflect true causation, for example, the presence of a psychiatric comorbidity was a significant protector of mortality in our cohort. But this could be the result of a lead-time bias, with improved recognition and documentation of these conditions in more recent years. It is also important to note that the study period overlapped with REDUCE trial [17•] at our centre—a feasibility study looking at the use of LTADs in RA. The study protocol mandated that patients get referred to PC which could have skewed our results and explains the strong association between LTAD insertion and PC referral. Additionally, a higher proportion may have received PC compared to other centres and our results might not be representative. Finally, as source of data was electronic letters rather than actual medical records, this may have resulted in some patients being inadvertently excluded. However, electronic records often do document information that clinicians find most important about patient care and is reflective of what community care teams have access to.

## Conclusions

RA is associated with high mortality with only a minority being eligible for curative options. There needs to be a multifaceted approach to improve the end-of-life experience of patients and their caregivers with advanced cirrhosis including those with RA. Our findings suggest patients with RA are denied timely access to PC services. More research into the optimal strategy and timing of interventions is needed to improve the holistic care needs of this cohort of patients.

## Supplementary Material

Supplementary Table 4

## Figures and Tables

**Table 1:** Results of scoping review showing studies assessing outcomes in refractory ascites

Study/country/publication year	Type of study and outcome	Sample size and RA definition	Demographics, baseline characteristics	Clinical outcomes	Data on palliative care	Other comments
Refractory hepatic hydrothorax (HHT) is an independent predictor of mortality when compared to refractory ascites (RA) Osman et al.. 2022 [23] USA	Retrospective 1:1 matched patients with RA vs. HHT based on age/gender and MELD-Na Primary outcome: comparison of mortality between the two groups	47 ‘Ascites that is either resistant/intractable to diuretics/low sodium diet, early recurrence of ascites. or has diuretic-induced complications’ HCC not excluded	Median age 59 (IQR 55–59) 26 (55%) male Median MELD-Na 18 (IQR 13–23.5) 51.2% alcohol aetiology 12 (25.3%) had previous SBP Ascitic protein not commented on	1-year mortality—19.2%, Median survival over> 12 months 2 (4.3%.) patients received TIPS Rates of transplant—unknown	No	Selected cohort of patients to match those with HHT—might not be truly representative
Outcomes and mortality of grade 1 ascites and recurrent ascites in patients with cirrhosis Tonon et al.. 2021 [45] Italy	Post hoc analysis of prospective cohort taking part in an outpatient management programme for over 14 years	56 International Ascites Club 1996 definition HCC excluded	Mean age 59 (± 10) 41 (73.2%) male Median MELD-Na 16 (IQR 13−21) 32 (57.1%) alcohol aetiology No comment on rates of SBP or ascitic fluid protein	13 (23.2%.) underwent liver transplant 26 (46.4%) died Median follow-up (for the entire cohort) was 29 months	No	Median survival NA RA vs. responsive ascites independent predictor of 36-month mortality. HR 3.60 (1.18–10.98)*p* =0.024
Frailty in nonalcoholic fatty liver cirrhosis: a comparison with alcoholic cirrhosis. risk patterns, and impact on prognosis Skladany et al.. 2021 [26] Slovakia	A cirrhosis registry investigating impact of frailty on clinical outcomes in NAFLD and ALD—a subset had RA	112 Definition of RA NA HCC not excluded	Mean age 58 ± 10 81 (72.3%) male 100 (89.2%) alcohol contributing aetiology Mean MELD-Na 19±6.8 SBP data or ascitic fluid protein NA	Median follow-up 9 months (2–18) 3 (2.7%) underwent transplant Median survival 10.3 months (CI interval 4.0–21.1) 1-year mortality 53.1%	No	Unpublished data, analysis done on raw data kindly provided by the authors Aetiology only including patients with ALD/NAFLD
Quality of life measures predict mortality in patients with cirrhosis and severe ascites Macdonald et al.. 2019 [24] Multinational randomised control trial	Retrospective evaluation of HRQoL data from published RCT assessing efficacy of satavaptan in ascites	241 Patients having 2 LVPs in the last 3 months on a sodium-restricted diet and having dose-limiting diuretic side effects HCC exceeding Milan criteria excluded	Median age 58 (IQR 51–66) 181 (75%) male 40 (17%9 had previous SBP 165 (68%) alcohol aetiology Median MELD 14 (IQR 11–18)	1-year mortality 29% (CI 23–35) 1-year follow-up 4 (1.7%) underwent a LT 2 (1.0%) underwent a TIPS 24 (12%) developed new onset SBP	No	Trial terminated due to increased mortality in one of the Satavaptan treatment group
Severe hyponatremia is a better predictor of mortality than MELDNa in patients with cirrhosis and refractory ascites (RA) Serste et al.. 2012 [25] France	Single-centre, observational. prospective study cirrhosis-related RA to establish predictors of mortality	174 Patients had to have at least 2 LVPs in 1 month. Minimum follow-up 3 months unless death. RA definition based on the IAC criteria HCC not excluded	Mean age 60.3 ± 11.6; 139 (79.9%) male Mean MELD-Na 22.8±4.4 96 (55.2%) alcohol aetiology Median ascitic fluid protein (11 g/L) Data on SBP NA	Median transplant free follow-up 8 months 34 (19.2%) underwent liver transplant 1-year mortality 55% (55-56)	No	Hyponatraemia (< 125) as the cause of RA and frequency of LVPs were independent predictors of morality (HR 2.11 *p* 0.001 and 1.42 *p* <0.0001)
Clinical characteristics and outcome of patients with cirrhosis and refractory ascites (RA) Moreau et ah. 2004 [8] France	One-year single-centre retrospective cohort study in patients with RA	75 Based on modified IAC definition HCC not specified as an exclusion criteria	Mean age 57 ± 9; 60 (80.5%)male 9 (12%) had previous SBP Mean ascitic fluid protein 15 (±7) g/L 35 (47%) Child-Pugh B disease Mean serum bilirubin, albumin, creatinine and prothrombin 45 (± 24) μmol/L; 28 (± 5) g/L; 112 (± 57) μmol/L and 53 (± 16) seconds, respectively	Mean follow-up 18 (± 13) months 1-year mortality 48%, (37–60) 8 (11%<) underwent liver transplant Age > 60 years commonest reason for transplant ineligibility—18 (26.7%)	No	Older age (50–60) compared to < 50. HR 4.4 (1.6–12). presence of HCC. HR 2.8 (1.0–4.8) and diabetes 2.2 (1.1 to 4.5) independent predictors of mortality, abstinence from alcohol protective. HR 0.34 (0.14 to 0.84)
Survival and prognostic factors and cirrhotic patients with ascites: a study of 134 outpatients Salerno et ah. 1993 [6] Italy	Prospective cohort study of 134 patients, a subset of which had developed refractory ascites	24 RA defined as progressively accumulating ascites (judged by body weight) and stable urinary sodium < 10 mmol/L despite diuretics at highest tolerated dose	Mean age 62 ± 2 Mean serum bilirubin, albumin and creatinine 2.7±0.4 mg/ dl. 3.1±0.1 g/dfand 1.3±0.1 mg/dl, respectively	3 (12.5%) underwent peritoneovenous shunt. 1 (4.2%) liver transplant and 2 (8.3%) on transplant wait list 1-year mortality 43%(KM curve)	No	Study > 30 years old prior to IAC definition of refractory ascites with small sample size

*RA* refractory ascites, *HCC* hepatocellular carcinoma, *SBP* spontaneous bacterial peritonitis, *TIPS* transjugular intrahepatic portosystemic shunt, *LVP* large-volume paracentesis, *IAC* International Ascites Club criteria, *HRQoL* health-related quality of life, *NAFLD* non-alcoholic fatty liver disease, *ALD* alcohol-related liver disease, *KM* Kaplan–Meier

**Table 2:** Baseline demographic and clinical data in study cohort

Age	59 ± 13 years
Gender (% Male)	69.3%
**Aetiology of cirrhosis** *	
Alcohol	71 (81%)
Metabolic associated liver disease	16 (18%)
Viral	8 (9%)
Other	7 (8%)
**Comorbidity**	
Cardiovascular	29 (33.0%)
Diabetes	28 (31.8%)
Mental health	21 (23.9%)
Respiratory	15 (17.1%)
Gastroenterological	12 (13.6%)
Non-HCC malignancy	14 (15.9%)
HCC	3 (3.4%)
Other	27 (30.7%)
On diuretics	60 (68.2%)
Previous SBP	33 (37.5%)
Prophylactic antibiotics	7 (8.0%)
Serum bilirubin (μmol/L)	29 (IQR 30.5)
Serum bilirubin > 51 μmol/L	21 (23.9%)
INR	1.3 (IQR 0.2)
INR > 1.2	47 (51%)
Serum creatinine (μmol/L)	71.5 (IQR 62.5)
Serum creatinine > 106 μmol/L	24 (27.3%)
Serum sodium (mmol/L)	131.8 (± 5.6)
Serum sodium < 128 mmol/L	18 (20.5%)
Serum albumin (g/L)	32.7 (± 5.2)
Serum albumin < 30 g/L	27 (30.7%)
Child-Pugh score	9 (IQR 8–10)
MELD-Na score	16.6 ± 6.9
UKELD score	55.7 ± 5.2
Ascitic fluid protein (g/L)	13 (IQR 9)
Ascitic fluid protein < 15 g/L	51 (58%)

* Note percentages add up to greater than 100% as several patients had multiple aetiologies for cirrhosis *HCC* hepatocellular cancer, *SBP* spontaneous bacterial peritonitis

**Table 3:** Outcomes in study cohort

Referred for liver transplant	31 (35%)
Underwent liver transplant	11 (13.6%)
**Reason why not referred**	
Comorbidity	17 (19.3%)
Alcohol/substance misuse/psychosocial issues	19 (21.6%)
Frailty	8 (9.1%)
Not specified	8 (9.1%)
Recompensated	3 (3.4%)
Unspecified MDT decision	2 (2.3%)
Referred for TIPS	6 (7%)
Underwent TIPS	1 (1%)
**Reasons why not referred**	
Substance misuse/psychosocial issues	11 (12.5%)
Serious comorbidity	12 (13.6%)
Hepatic encephalopathy	13 (14.8%)
Frailty	10 (11.4%)
Unspecified MDT decision	1 (1.1%)
Not specified	20 (22.7%)
Recompensating/improvement of refractory ascites	6 (6.8%)
Referred for transplant/listed	8 (9.1%)
Advanced liver disease	1 (1.1%)
Median number of large-volume paracentesis	5 (IQR 3–8)

*MDT* multi-disciplinary team, *LTAD* long-term ascitic drain, *TIPS* transjugular intrahepatic portosystemic shunt

**Table 4:** Palliative care input

Documented end of life discussion	50 (56%)
Referred to palliative care	29 (33%)
Weeks between diagnosis and referral to palliative care	15 (IQR 4–32)
LTAD inserted	11
Received prophylactic antibiotics after LTAD	8

*LTAD* long-term abdominal drain
