# Supplementary material for: Outcomes in Cirrhosis-Related Refractory Ascites with Emphasis on Palliative Care: Single-Centre Experience and Literature Review
Source: Curr Hepatol Rep. Author manuscript; Available in PMC 2025 Nov 4. (PMC7618328; doi:10.1007/s11901-024-00669-0)
Supplement: Supplementary Table 4 [file EMS209527-supplement-Supplementary_Table_4.pdf]

Table 4 (Supplementary) – Univariate & multivariate analysis of predictors of mortality in study cohort

	Univariate analysis			Multivariate analysis		
**Key Variable**	**OR**	**CI**	**P value**	**OR**	**CI**	**P value**
Age (per year)	1.04	1.01-1.08	**0.020***	1.03	0.99-1.08	0.095
Male	1.77	0.70-4.44	0.225			
Child Pugh Score C	0.66	0.26-1.71	0.394			
MELD-Na	1.04	0.98-1.11	0.232			
UKELD	1.01	0.93-1.09	0.892			
History of SBP	0.63	0.26-1.53	0.310			
Continued diuretics	0.66	0.26-1.71	0.394			
Gastrointestinal comorbidity	1.30	0.36-4.72	0.685			
Cardiovascular comorbidity	2.65	0.98-7.16	0.054			
Respiratory comorbidity	1.92	0.56-6.60	0.301			
Psychiatric comorbidity	0.28	0.10-0.78	**0.015***	0.32	0.11-0.97	**0.044**
Renal comorbidity	4.12	0.47-35.9	0.199			
Neurological comorbidity	0.83	0.17-3.94	0.811			
Endocrine comorbidity	5.74	0.68-48.1	0.107			
Malignancy	1.16	0.35-3.81	0.807			
TIPS/Transplant	0.16	0.04-0.66	**0.011***	0.15	0.04-0.64	**0.010**
LTAD insertion	1.78	0.44-7.31	0.413			

Bases for all categorical variables are the ‘no’ category or absence of comorbidity, for Child Pugh Score C the base is Child Pugh Score B. TIPS Trans jugular intrahepatic portosystemic shunt; LTAD long-term abdominal drain
